# Ensiling Time and Mixed Microbe Fermented Liquid Modulate In Vitro Digestibility and Rumen Fermentation of Fermented Total Mixed Rations

**DOI:** 10.3390/vetsci13010006

**Published:** 2025-12-20

**Authors:** Sineenart Polyorach, Wichai Suphalucksana, Ampon Klompanya, Chalermpon Yuangklang, Metha Wanapat, Seangla Cheas, Anusorn Cherdthong, Sungchhang Kang, Pongsatorn Gunun, Nirawan Gunun, Suban Foiklang, Phongthorn Kongmun, Nattaya Montri, Kanokrat Srikijkasemwat

**Affiliations:** 1 Department of Animal Production Technology and Fisheries, School of Agricultural Technology, King Mongkut’s Institute of Technology Ladkrabang, Bangkok 10520, Thailand; kswichai@kmitl.ac.th (W.S.); ampon.kl@kmitl.ac.th (A.K.); kanokrat.sr@kmitl.ac.th (K.S.); 2 Department of Animal Science, Faculty of Agricultural Innovation and Technology, Rajamangala University of Technology Isan, Main Campus, Nakhon Ratchasima 30000, Thailand; chalermpon.yu@rmuti.ac.th; 3 Tropical Feed Resources Research and Development Center (TROFREC), Department of Animal Science, Faculty of Agriculture, Khon Kaen University, Khon Kaen 40002, Thailand; metha@kku.ac.th (M.W.); seangla.c@kkumail.com (S.C.); anusornc@kku.ac.th (A.C.); 4 Agricultural Unit, Department of Education, National Institute of Education, Phnom Penh 12200, Cambodia; ksungchhang@yahoo.com; 5 Department of Animal Science, Faculty of Natural Resources, Rajamangala University of Technology Isan, Sakon Nakhon Campus, Phangkhon, Sakon Nakhon 47160, Thailand; pongsatorn.gu@rmuti.ac.th; 6 Department of Animal Science, Faculty of Technology and Engineering, Udon Thani Rajabhat University, Udon Thani 41000, Thailand; nirawan.gu@udru.ac.th; 7 Faculty of Animal Science and Technology, Maejo University, Chiang Mai 50290, Thailand; suban@mju.ac.th; 8 Department of Animal Science, Faculty of Agriculture, Kasetsart University, Bangkok 10900, Thailand; 9 Office of Administrative Interdisciplinary Program on Agricultural Technology, School of Agricultural Technology, King Mongkut’s Institute of Technology Ladkrabang, Bangkok 10520, Thailand; nattaya.mo@kmitl.ac.th

**Keywords:** fermented total mixed ration, in vitro gas production, methane mitigation, mixed microbes fermented liquid, rumen fermentation

## Abstract

This study investigated how varying the ensiling period and the level of mixed microbes fermented liquid (MMFL) influences the chemical composition, in vitro degradability, rumen fermentation profile, and microbial ecology of fermented total mixed rations (FTMR). A completely randomized 4 × 4 factorial design was used, incorporating four fermentation durations (0, 7, 14, and 21 days) and four MMFL inclusion rates (0, 0.5, 1.0, and 1.5% of DM). Both factors exerted significant effects on FTMR quality (*p* < 0.05). The combination of a 14-day fermentation period with 0.5% MMFL consistently produced the most desirable outcomes. Under these conditions, crude protein concentration rose from 12.0% to 14.3% of DM, while neutral-detergent fiber declined from 54.2% to 49.1%. Improvements were also observed in in vitro digestibility, with DM and OM increasing by 9–12% relative to the untreated control (*p* < 0.05). Fermentation end-products were enhanced, as total volatile fatty acids increased by 15% (92.4 vs. 80.1 mmol/L), and the molar proportion of propionate increased from 24.5 to 29.2 mol/100 mol, thereby lowering the acetate-to-propionate ratio (2.4 vs. 3.0; *p* < 0.05). Estimated methane production declined by 18% (*p* < 0.01). Microbial counts reflected a shift toward a more efficient fermentative community, with bacterial and fungal populations increasing by 21% and 18%, and protozoa decreasing by 25% (*p* < 0.05). Overall, moderate MMFL supplementation during a 14-day ensiling phase enhanced nutrient conservation and fermentation efficiency, suggesting practical value for improving FTMR utilization in ruminant systems. Further in vivo and economic evaluations remain necessary.

## Simple Summary

Fermented total mixed rations (FTMR) can enhance feed stability and utilization, but their performance varies with ensiling duration and microbial enrichment. This study evaluated FTMR treated with mixed microbes fermented liquid (MMFL) using in vitro gas production assays. A 14-day fermentation combined with 0.5% MMFL improved nutrient profiles, increased digestibility, and boosted volatile fatty acid formation while lowering fiber content and estimated methane output. Microbial communities shifted toward higher bacterial and fungal populations and fewer protozoa. These results indicate that MMFL is a promising additive for producing higher-quality FTMR and supporting more efficient, sustainable ruminant systems.

## 1. Introduction

Fermented total mixed ration (FTMR) refers to a total mixed ration preserved by anaerobic fermentation before feeding. This feeding approach has gained attention because it improves nutrient preservation and feed stability. During fermentation, pH declines and lactic acid accumulates, restricting spoilage organisms and extending storability while improving the breakdown of fibrous and protein-rich components [1]. Feeding FTMR also encourages the proliferation of beneficial lactic acid–producing microbes, which helps limit mold and yeast growth and improves aerobic stability after silo opening [2]. These features make FTMR suitable for sustaining feed consistency and animal performance across variable tropical production systems.

Previous work indicates that FTMR can enhance dry matter (DM) intake (5–12%), organic matter (OM) digestibility (4–10%), and microbial protein synthesis, leading to measurable improvements in growth performance and milk yield [1,3]. Incorporating crop residues such as cassava pulp or corn stover into FTMR is particularly attractive in tropical regions because these materials are abundant, low-cost, and highly fermentable when combined with readily available carbohydrate sources [4]. The application of microbial inoculants—such as lactic acid bacteria (LAB) or exogenous enzymes—has further been reported to reduce proteolysis, stabilize nutrient composition, and improve overall fermentation quality [5]. Additionally, several studies demonstrate that FTMR has been reported to influence overall rumen microbial activity and fermentation patterns, as reflected by changes in total microbial populations and fermentation end-products [6]. Collectively, these effects highlight the value of FTMR in improving feed efficiency and supporting a more favorable rumen microbial environment. Beyond the general benefits of FTMR, the effectiveness of the fermentation process is increasingly linked to the type and complexity of microbial inoculants applied during ensiling [2,4]. In contrast to traditional single-strain additives, mixed microbial formulations have recently attracted interest because they combine complementary microbial functions that may enhance fermentation stability and nutrient preservation.

Within this context, the success of FTMR depends strongly on the microbial additives used during ensiling. Mixed cultures containing *Saccharomyces cerevisiae* and effective microorganisms (EMs) such as *Lactobacillus plantarum* and *Rhodopseudomonas palustris* have been reported to enhance protein enrichment, improve fermentation efficiency, and maintain aerobic stability [7]. Mixed microbes fermented liquid (MMFL)—a combination of LAB, yeasts, and *Bacillus* spp.—has recently drawn interest because of its synergistic actions during fermentation. Supplementation with MMFL has been shown to increase lactic acid production, hasten pH decline, and suppress undesirable microbes, thereby improving nutrient preservation [8]. These microbial shifts may alter rumen fermentation patterns, particularly the acetate-to-propionate balance. Acetate formation is associated with greater hydrogen release, whereas propionate serves as a competing hydrogen sink and can reduce enteric methane production. For this reason, assessing fermentation end-products, including estimated methane output, is important for understanding both nutritional and environmental outcomes. Earlier studies reported that MMFL increases crude protein (CP), reduces fiber fractions, and limits ammonia-nitrogen (NH_3_-N) accumulation, indicating improved feed quality [7,8,9]. High concentrations of structural fiber are known to restrict microbial access to substrates and reduce digestibility, thereby limiting nutrient utilization. Reductions in fiber content have been associated with improved fermentation efficiency and in vitro digestibility in forages and silages [7,8]. In addition, MMFL has been shown to promote beneficial microbes such as *Lactobacillus* while suppressing undesirable organisms, contributing to better feed stability and nutritive value [10].

Additional evidence from studies using microbial fermented liquid (MFL) supports these findings. In vitro experiments and trials in early lactating cows showed that including MFL at 20% of the concentrate increased total gas production and fermentation rate. Although higher gas production can indicate enhanced microbial activity, it may also reflect less efficient fermentation; in these studies, it was accompanied by improved digestibility and higher milk yield [9,11]. Similarly, yeast- or EM-fermented cassava peel replaced up to half of the concentrate in goat diets without reducing feed intake, digestibility, or growth, and lowered feed cost per unit of gain by 32% [12]. These results indicate that microbial consortia can play an important role in enhancing fermentation processes and improving overall production efficiency.

Although these benefits are promising, the performance of FTMR is influenced by the duration of fermentation as well as the level of additive used [1]. Conventionally prepared total mixed rations (TMR) often suffer from greater proteolysis, nutrient losses, and limited fiber digestibility when ensiled without microbial inoculants [2,11]. While single-strain inoculants have been examined, less information is available on the combined effects of MMFL and different fermentation times in FTMR [9]. Evidence is still limited on how these factors interact to shape chemical composition, degradability, rumen fermentation responses, microbial populations, and methane production.

Most previous studies have emphasized individual inoculants such as LAB or yeast, but interactions among multi-strain microbial consortia during ensiling remain inadequately characterized under tropical feeding conditions. This knowledge gap is particularly relevant for tropical systems, where rapid spoilage and variable raw materials can affect fermentation.

Digestibility can be evaluated using in vivo or in vitro techniques. In vivo methods provide direct estimates but require substantial resources, whereas in vitro approaches allow controlled and repeatable comparison of multiple treatments. Gas production techniques are well suited for assessing fermentative degradation and rumen fermentation responses among diets [7], while marker-based methods are mainly applied in in vivo studies.

Therefore, the objective of the present study was to investigate how fermentation time and MMFL supplementation influence the chemical composition, in vitro gas production kinetics, rumen fermentation characteristics, estimated methane output, and microbial populations of FTMR. We hypothesized that selecting an appropriate ensiling duration together with moderate MMFL supplementation would enhance nutrient preservation, improve fermentation efficiency, and reduce methane estimation under in vitro conditions.

## 2. Materials and Methods

All procedures involving animals were reviewed and approved by the Animal Ethics Committee of King Mongkut’s Institute of Technology Ladkrabang in accordance with the guidelines of the Animal Care and Use Committee (Approval No. ACUC-KMITL-RES/2023/003).

### 2.1. Preparation of Microbial Mixed Fermented Liquid (MMFL)

Mixed microbes fermented liquid was prepared following Polyorach et al. [9] with slight modification. The effective microorganisms (EMs) were obtained directly from Bionova Biogas GmbH, Am Großen Zug 24a, 15713 Königs Wusterhausen, OT Wernsdorf, Germany (https://bionova-biogas.de/en/) (accessed on 10 October 2025). The product was purchased in June 2023 and used immediately after activation. According to the manufacturer, it contained approximately 1.3 × 10^7^ CFU/mL lactic acid bacteria, 3.3 × 10^4^ CFU/mL photosynthetic bacteria, and 1.3 × 10^4^ CFU/mL yeasts. Although the urea concentration in solution B (48 g per 100 mL) appears high, it was used as a concentrated nitrogen source prior to dilution. After mixing solutions A and B at a 1:1 ratio, the effective urea concentration was reduced by half, and the activated MMFL was subsequently applied at low inclusion levels (0.5–1.5% of diet DM). Similar high-nitrogen activation strategies have been reported in effective microorganism and fermented liquid systems, where urea serves as a rapidly available nitrogen source to stimulate microbial growth without causing toxicity when appropriately diluted [9,12]. Nevertheless, the potential sensitivity of certain microbial groups to high nitrogen environments cannot be excluded and represents a limitation of the present preparation method. Solutions A and B were then mixed at a 1:1 ratio and incubated at room temperature (25 °C) for 48 h. The fermented liquid obtained after incubation was used as MMFL for the experiment. All chemical analyses were performed in duplicate to ensure accuracy, and data were expressed on a DM basis. Mixed microbes fermented liquid was freshly prepared for each fermentation batch to maintain microbial viability and consistency among treatments. The total mixed ration (TMR) was thoroughly mixed with the assigned level of MMFL and packed into laboratory-scale polyethylene silo bags (approximately 10 kg fresh matter per bag). The material was manually compacted to minimize air entrapment, sealed tightly, and stored under anaerobic conditions at ambient room temperature (25–30 °C). Fermentation durations were 0, 7, 14, and 21 days, according to the experimental design. After each ensiling period, the silos were opened, and representative samples were collected for chemical analysis and subsequent in vitro incubation.

Fermentation characteristics such as pH and organic acid concentrations (lactic and acetic acids) were not measured during or after ensiling. Therefore, changes in chemical composition were interpreted based on nutrient analysis and in vitro fermentation responses rather than direct silage fermentation indices. Samples of rice straw, TMR ingredients, and FTMR were analyzed for proximate composition before use in the experiment. Dry matter, OM, CP, and ether extract (EE) were determined following the procedures of AOAC [13]. Neutral detergent fiber (NDF) and acid detergent fiber (ADF) were determined according to the detergent system of Van Soest et al. [14], with sodium sulfite and α-amylase included in the NDF assay. All chemical analyses were performed in duplicate, and results were expressed on a DM basis.

### 2.2. Experimental Design and Treatments

The experiment followed a 4 × 4 factorial arrangement within a completely randomized design (CRD). Factor A represented the fermentation duration of the total mixed ration (0, 7, 14, and 21 days), and Factor B represented the level of MMFL supplementation at 0, 0.5, 1.0, and 1.5% of diet dry matter (DM). This factorial combination resulted in 16 treatment groups in total. A factorial design was selected to allow simultaneous evaluation of the main effects of fermentation duration and MMFL supplementation level, as well as their interaction. Fermentation responses in FTMR are expected to depend not only on additive dose or ensiling time alone, but also on how these factors interact during microbial succession and substrate utilization. The 4 × 4 factorial arrangement therefore provides a more robust framework for identifying optimal combinations and detecting non-additive responses that would not be captured in separate single-factor trials. The treatment without MMFL addition (0% MMFL) and with 0 days of fermentation served as the control group, representing the unfermented TMR baseline. Table 1 presents the formulation and proximate composition of the unfermented TMR, including rice straw as the primary roughage component.

### 2.3. Animals and Preparation of Rumen Inoculum

Two rumen-fistulated dairy steers (approximately 2 years old, 350 ± 30 kg body weight) were obtained from the Animal Research Unit, Faculty of Agricultural Technology, King Mongkut’s Institute of Technology Ladkrabang (KMITL), Thailand. Rumen fluid collected from both animals was pooled to minimize individual animal variation and to obtain a representative inoculum, which is commonly applied in controlled in vitro fermentation studies. The animals were clinically healthy and maintained under standard management conditions. Breeder consent and institutional approval for animal use were obtained in accordance with the Animal Ethics Committee of KMITL (Approval No. ACUC-KMITL-RES/2023/003). Animals were housed in individual pens with free access to clean water and mineral blocks. They were fed ad libitum with a rice straw–based FTMR formulated to contain 10.5% CP and 65.6% total digestible nutrients. This adaptation diet did not contain MMFL or any other microbial inoculants and followed the standard feeding program of the animal research unit. Feed was provided twice daily at 07:00 and 16:00 h. Prior to inoculum collection, steers were adapted to the experimental diet for 21 days. On day 21, approximately 1000 mL of rumen fluid was collected before the morning feeding. Fluid was filtered through four layers of cheesecloth, transferred into pre-warmed thermos flasks, and transported to the laboratory under anaerobic conditions for immediate use in in vitro incubations.

### 2.4. In Vitro Fermentation Procedure

The substrate-to-inoculum ratio was maintained at 1:150 (g DM: mL buffered rumen fluid), following the method of Nampukdee et al. [11]. Each FTMR treatment sample (200 mg DM) was weighed into 50 mL serum bottles. Rumen fluid collected from both steers was pooled in equal proportions and mixed with artificial saliva solution [11] at a 2:1 (*v*/*v*) ratio under continuous CO_2_ flushing to ensure anaerobic conditions. Thirty milliliters of this buffered inoculum were dispensed into each bottle, which was immediately sealed with butyl rubber stoppers and aluminum crimps to maintain airtightness. Bottles were incubated in a shaking water bath (used as the incubator) at 39 ± 0.5 °C for 96 h to simulate rumen temperature and promote uniform mixing. Gas pressure was measured at predetermined intervals up to 72 h for kinetic modeling, and incubation was continued to 96 h for determining in vitro DM and OM degradability. After each pressure reading, bottles were briefly vented through the septum to avoid overpressure and ensure accurate gas volume estimation. Each treatment was conducted in four replicates within each in vitro incubation run, and the experiment was performed across four independent runs. Each incubation bottle was considered the experimental unit for statistical analysis. Blank bottles containing only inoculum were included in each run. Net gas production was calculated by subtracting blank values from the corresponding treatment readings.

### 2.5. Gas Measurement and Kinetics

Cumulative gas production was recorded at 0, 2, 4, 6, 8, 12, 16, 24, 48, and 72 h of incubation using a pressure transducer connected to a calibrated glass syringe. The recorded gas volumes were then fitted to the non-linear exponential model of Ørskov and McDonald [15]:

Y = *a* + *b* (1 − e^−*ct*^)

where Y represents the cumulative gas production (mL) at time t (h), a is the gas production from the immediately soluble fraction, b is the gas production from the insoluble but fermentable fraction, and c is the fractional rate constant of gas production per hour. The sum (a + b) indicates the potential extent of gas production. Model parameters (a, b, and c) were estimated using the non-linear regression procedure (PROC NLIN) in SAS (Version 9.4), and the goodness of fit was assessed by examining the coefficient of determination (R^2^) and residual distribution to ensure model accuracy.

At 12 h of incubation, rumen fluid samples were collected and filtered through cheesecloth. Fermentation parameters were determined at 12 h to capture the phase of maximum microbial activity, as commonly adopted in short-term in vitro trials. We recognize that multi-time-point sampling would better describe fermentation dynamics and will be considered in future work. The first portion of the filtrate was centrifuged at 16,000× *g* for 15 min, and the supernatant was stored at −20 °C for subsequent analysis. Ammonia-nitrogen (NH_3_-N) concentration was determined by the micro-Kjeldahl method [13]. Each chemical and volatile fatty acids (VFA) analysis was performed in duplicate, and mean values were used for statistical evaluation. Volatile fatty acids were quantified by high-performance liquid chromatography (HPLC) using a Rezex ROA Organic Acid H^+^ column (300 × 7.8 mm, Phenomenex, Torrance, CA, USA). The mobile phase consisted of 0.005 N H_2_SO_4_ at a flow rate of 0.6 mL/min, and detection was performed with a UV detector at 210 nm. Individual VFAs (acetate, propionate, and butyrate) were identified and quantified by comparing their retention times and peak areas with those of analytical standards. Calibration curves were constructed using standard solutions of known concentrations, and VFA concentrations in the samples were calculated based on the corresponding peak areas using the HPLC (Shimadzu LabSolutions software version 5.110; Shimadzu Corporation, Kyoto, Japan). Total VFA concentration was obtained by summing the individual acid concentrations. Methane production was estimated using the stoichiometric equation of Moss et al. [16]: CH_4_ = (0.45 × acetate) − (0.275 × propionate) + (0.40 × butyrate). Individual VFA concentrations (acetate, propionate, and butyrate) were expressed in mmol/L and calculated from total VFA concentration and their respective molar proportions. Estimated methane values are therefore reported as mmol/L of incubation fluid and represent relative indicators of methanogenic potential rather than directly measured methane production.

A second portion of rumen fluid was fixed with 10% formalin in sterile 0.9% saline solution for enumeration of microbial populations. Total bacteria, protozoa, and fungal zoospores were counted using a hemocytometer under a light microscope according to Martins et al. [17]. Bacterial counts were performed after staining with methylene blue, protozoa were identified and counted using Lugol’s iodine solution, and fungal zoospores were enumerated with lactophenol cotton blue stain.

In vitro degradability was determined after 96 h of incubation. Digestibility was measured after 96 h incubation to represent near-complete fermentation under in vitro conditions, which typically extends beyond the 24–48 h in vivo rumen passage time. Fermentation residues were filtered through pre-weighed Gooch crucibles, and the weight loss was used to calculate in vitro DM degradability (IVDMD). Residues were then ashed at 550 °C to determine in vitro OM degradability (IVOMD) following the method of Tilley and Terry [18].

### 2.6. Statistical Analysis

Data were examined for normality (Shapiro–Wilk) and homogeneity of variance (Levene’s test) before analysis. All data were analyzed as a 4 × 4 factorial arrangement in a completely randomized design (CRD) using the PROC GLM procedure of SAS [17]. Incubation bottles were treated as experimental units, with replicate bottles nested within runs. The statistical model was:

Yij = μ + Ai + Bj + (AB)ij + εij

where Yij is the observation, μ is the overall mean, Ai is the effect of fermentation time (i = 1–4), Bj is the effect of MMFL supplementation level (j = 1–4), (AB)ij is the interaction effect between fermentation time and MMFL level, and εij is the residual error. When treatment effects were significant (*p* < 0.05), mean comparisons were conducted using Tukey’s Honest Significant Difference (HSD) test. In addition, orthogonal polynomial contrasts were applied to examine linear, quadratic, and cubic responses to fermentation time and MMFL inclusion levels.

## 3. Results

### 3.1. Chemical Composition of Diet Ingredients and FTMR

Rice straw used as the main forage source contained 92.1% DM, 82.7% OM, 3.2% CP, and 74.8% NDF on a DM basis (Table 1). The formulated total mixed ration (TMR) provided 60.5% DM, 89.8% OM, 12.0% CP, 3.0% EE, 54.2% NDF, and 31.0% ADF. The chemical composition of the FTMR after fermentation is shown separately in Table 2 to demonstrate changes in nutrient composition resulting from MMFL inclusion and fermentation duration. The FTMR treatment fermented for 0 days with 0% MMFL represented the unfermented control, serving as the baseline composition for comparison with other fermentation times and supplementation levels. Increasing fermentation time significantly decreased NDF and ADF contents (*p* < 0.05), while CP concentration increased, particularly at 14 and 21 days of ensiling. Supplementation with MMFL elevated CP and reduced fiber fractions in a dose-dependent manner (*p* < 0.05). A significant interaction was observed between fermentation time and MMFL level, where the highest CP and lowest fiber contents occurred at 14 days with 0.5% MMFL supplementation.

### 3.2. Gas Production Kinetics and In Vitro Degradability

Slight irregularities in IVDMD values at day 0 likely resulted from inherent variability in inoculum activity rather than treatment effects. Gas production kinetics were influenced by both fermentation time and MMFL supplementation level (Table 3). The negative value of parameter a at the starting point (0 h) resulted from the curve-fitting process of the Ørskov and McDonald [15] model. This value reflects the initial microbial lag phase before fermentation begins and does not represent actual negative gas production. The rapidly soluble fraction (a) and the potential extent of gas production (a + b) increased with longer fermentation time (*p* < 0.05). Mixed microbes fermented liquid supplementation also enhanced a and a + b values, with 0.5% inclusion showing the greatest response. The insoluble fraction (b) and the rate constant (c) showed variable responses but were generally improved at 14 days of ensiling with MMFL addition. Gas volume increased steadily over time and was consistently higher in FTMR treated with MMFL than in the control. After 96 h of incubation, both IVDMD and IVOMD values were significantly higher in FTMR subjected to 14 days of fermentation and supplemented with 0.5% MMFL (*p* < 0.05).

### 3.3. Rumen Fermentation Characteristics

Total VFA concentration was significantly affected by both fermentation time and MMFL inclusion (*p* < 0.05) (Table 4). The highest values were observed in FTMR ensiled for 14 days with 0.5% MMFL. Acetate proportion decreased slightly with MMFL supplementation, while propionate increased, leading to a lower acetate-to-propionate ratio (*p* < 0.05). Butyrate levels were not consistently altered by treatments. Ammonia nitrogen concentration increased with fermentation time and MMFL addition (*p* < 0.05). Methane yield estimated from VFA profiles declined progressively with MMFL supplementation, with the lowest values detected in the 0.5% MMFL treatment at 14 days of fermentation (*p* < 0.05).

### 3.4. Rumen Microbial Populations

Rumen microbial populations were influenced by both main factors and their interaction (Table 5). Total bacterial counts increased with longer fermentation time and with MMFL inclusion, peaking at 14 days with 0.5% supplementation (*p* < 0.05). Protozoal counts declined significantly with MMFL addition (*p* < 0.05), and the lowest counts were observed in FTMR fermented for 14 days with 0.5% MMFL. Fungal zoospore populations increased under the same conditions, suggesting that both fermentation duration and MMFL level influenced microbial community balance.

## 4. Discussion

A limitation of this study is that the final characteristics of the MMFL after 48 h of activation, including pH, organic acid composition, and viable microbial counts, were not determined. These parameters are important indicators of microbial activity and fermentation potential because pH and organic acid profiles reflect the extent of acidification, while viable microbial counts indicate the capacity of the inoculant to actively participate in fermentation [1,2]. Variation in these characteristics from the original commercial EM formulation may influence fermentation rate, nitrogen preservation, and fiber degradation, potentially affecting the consistency and magnitude of responses observed among treatments [7,9]. Fermentation time and MMFL supplementation independently and interactively improved the nutritive value of FTMR. Extended ensiling increased CP and ether extract while reducing fiber fractions, indicating enhanced nutrient preservation and structural carbohydrate degradation. Part of the observed increase in CP may be associated with nitrogen supplied by MMFL, although the relative contribution of nitrogen input versus microbial protein synthesis during fermentation could not be distinguished in the present study. Similar responses occurred with MMFL addition, which elevated protein and energy fractions and lowered NDF and ADF contents in the present study. However, lactic acid production and pH reduction were not directly measured. Therefore, comparisons with earlier studies reporting enhanced lactic acid formation and pH decline following mixed inoculant use are made at a conceptual level rather than as direct observations [9].

Extended fermentation in this study was associated with more stable CP levels, which parallels previous reports showing that prolonged ensiling with additives such as *Lactobacillus plantarum* and molasses can limit proteolysis in alfalfa-based FTMR [19]. The improvement in CP retention with MMFL supplementation may reflect synergistic interactions among lactic acid bacteria, yeast, and *Bacillus* spp., which have been shown to reduce proteolysis and support protein stabilization during ensiling [20].

Heterofermentative lactic acid bacteria further alter fermentation pathways, limiting nutrient losses and favoring retention of soluble nitrogenous compounds [8]. This microbial synergy preserves protein and supports partial fiber degradation. Fibrolytic enzymes, including cellulases and hemicellulases, increase cell wall accessibility and facilitate controlled carbohydrate breakdown. Similar outcomes have been reported where MMFL supplementation increased apparent digestibility of CP, NDF, and ADF in ruminants [9]. Additives combining lactic acid bacteria and cellulase also increase CP content and silage quality, leading to improved digestibility [21]. Collectively, these findings suggest that the observed changes in FTMR composition arise from microbial degradation and preservation mechanisms during ensiling.

Gas production and degradability patterns reflected the combined influence of fermentation duration and MMFL level. Gas production potential (a + b) and in vitro digestibility increased progressively up to 14 days, then declined with longer fermentation. This indicates that 14 days of ensiling provides optimal microbial stabilization and nutrient preservation. In contrast, excessive fermentation duration may promote carbohydrate depletion, increased proteolysis, and secondary fermentation, leading to nutrient losses. Similar trends have been observed where mid-length fermentation balanced microbial growth and substrate conservation, enhancing digestibility while limiting spoilage [22]. At this stage, lactic acid bacteria dominate the microbial community, producing sufficient lactic acid to reduce pH and stabilize silage [23]. This stabilization supports improved digestibility of OM through enhanced breakdown of complex carbohydrates [24]. Shorter fermentation periods often result in incomplete microbial stabilization and higher spoilage risk, while very long ensiling promotes over-fermentation and nutrient loss [25]. Mixed microbes fermented liquid supplementation further improved degradability, with the most consistent response observed at 0.5%. At 1.5% MMFL, digestibility and gas production declined. This response may be associated with excessive microbial activity or accumulation of fermentation end-products, such as organic acids or ammonia, which have been reported to inhibit microbial efficiency when present at high concentrations during ensiling and rumen fermentation [1]. The effect is attributed to the introduction of beneficial microbial consortia that enhance enzymatic activity, improve microbial balance, and suppress undesirable organisms [26]. Moderate MMFL levels support microbial synergy, increasing DM and OM digestibility [11], while excessive inclusion (1.5%) may disrupt microbial equilibrium, resulting in reduced efficiency.

In this study, only the total viable microbial counts were determined, without differentiation among specific microbial groups. Therefore, the discussion was limited to the overall activity of EM present in the mixed microbial inoculum rather than individual taxa.

Rumen fermentation characteristics confirmed the role of fermentation time and MMFL in shaping end-products. The highest total VFA concentration and propionate proportion occurred in FTMR ensiled for 14 days with 0.5% MMFL supplementation, whereas acetate proportion, acetate-to-propionate ratio, and methane yield declined. These shifts demonstrate improved fermentation efficiency and a more glucogenic profile, supporting energy supply for ruminants. Similar alterations in VFA profiles have been associated with functional shifts in rumen fermentation favoring propionate production. However, in the present study, these microbial shifts are inferred from fermentation end-products rather than directly measured, as molecular identification of specific microbial taxa was not performed [27]. Previous FTMR studies have reported associations between lactic acid bacteria activity and increased propionate formation, although such taxonomic changes were not directly assessed in the present study [6]. Propionate serves as a direct substrate for hepatic gluconeogenesis, enhancing glucose supply for production animals, while acetate is closely associated with methanogenesis. Thus, a reduction in the acetate-to-propionate ratio is commonly associated with lower methanogenic potential and may contribute to reduced methane formation under in vitro conditions [27]. High VFA concentrations may also inhibit methanogens, as acidic conditions suppress their growth and activity [28]. VFAs themselves, particularly acetate and propionate, have been reported to exert inhibitory effects on methanogenesis [29]. These findings indicate that optimal fermentation and MMFL supplementation not only enhance energy efficiency but also contribute to environmental sustainability through reduced methane estimation. Because methane output was estimated using a stoichiometric model based on VFA proportions [16] rather than measured directly, the results should be interpreted as relative indicators of methanogenic potential. This approach assumes a fixed relationship between VFA formation and hydrogen balance and does not account for alternative hydrogen sinks or variability in microbial pathways, which introduces inherent uncertainty. Another limitation of the present study is the absence of single-strain inoculant controls, which restricts the ability to distinguish the individual contributions of microbial groups within MMFL. Future studies should incorporate both positive controls (single microbial inoculants) and negative controls to better resolve the specific roles and synergistic interactions of lactic acid bacteria, yeasts, and *Bacillus* species in FTMR fermentation and rumen responses

References to specific microbial taxa (e.g., Prevotella, Ruminococcus, and Methanobrevibacter) are hypothetical and based on patterns reported in similar FTMR studies, as molecular identification was not performed here. Rumen microbial populations reflected the shifts observed in fermentation efficiency. Bacterial and fungal populations increased with 14 days of fermentation and MMFL supplementation, whereas protozoal counts declined under the same conditions. This pattern suggests enhanced fibrolytic activity and reduced protozoal-driven nitrogen recycling [30]. FTMR fermentation increased total bacterial and fungal populations, suggesting enhanced fibrolytic and fermentative activity, although microbial diversity and taxonomic composition were not determined [6]. A concurrent decline in protozoa, particularly *Ostracodinium*, supports improved microbial protein synthesis by reducing nitrogen turnover through protozoal predation. Increased fungal richness, particularly within Ascomycota and Basidiomycota, further suggests enhanced fibrolytic potential, while reductions in opportunistic fungi such as *Candida* and *Cryptococcus* may result from antifungal metabolites produced by beneficial inoculants, including *Lactobacillus acidophilus* and *Bacillus subtilis* [31]. The synergistic actions of lactic acid bacteria and yeast explain much of this response. Lactic acid bacteria reduce silage pH and stabilize fermentation, whereas yeast helps maintain rumen pH and stimulates fibrolytic bacteria. Together, these effects enhance acetate, propionate, and butyrate production [32,33]. This synergy improves nutrient utilization, as yeast promotes cellulolytic activity and lactic acid bacteria enhance carbohydrate metabolism while suppressing pathogens [34,35]. Shifts toward genera such as *Prevotella* and *Ruminococcus* also support butyrate production, which strengthens rumen epithelial integrity and further promotes fermentation efficiency [36,37].

The overall pattern of gas production and degradability observed in this study indicates that both fermentation duration and MMFL inclusion influenced microbial fermentation efficiency. The increase in gas production potential (a + b) and digestibility up to 14 days of fermentation followed by a decline thereafter suggests that microbial activity reached its optimum around mid-fermentation. This response is likely due to the synergistic action of mixed EM such as Lactobacillus, Saccharomyces, and Bacillus spp., which enhance fiber degradation and promote favorable fermentation conditions within the FTMR [38,39,40]. Although the present work did not quantify individual microbial populations or directly measure methane, these limitations do not alter the interpretation that EM supplementation effectively enhanced rumen fermentation dynamics under in vitro conditions.

## 5. Conclusions

Fermentation time and MMFL supplementation improved the chemical composition, degradability, and rumen fermentation characteristics of FTMR. Ensiling for 14 days with 0.5% MMFL consistently produced the most favorable responses, including higher crude protein content, improved digestibility, increased propionate production, reduced protozoal populations, and lower estimated methane yield. These results indicate that MMFL is a promising additive for enhancing FTMR quality and fermentation efficiency under in vitro conditions. Further in vivo studies are required to confirm the effects on animal performance, nitrogen utilization, and methane emissions under practical feeding conditions. In addition, aerobic stability of the optimal 14-day FTMR supplemented with 0.5% MMFL should be evaluated, as resistance to spoilage after silo opening is a critical factor for fermented feeds in warm and humid tropical environments.

## Figures and Tables

**Table 1:** Ingredients and chemical composition of total mixed ration (TMR) and rice straw in the experiment.

Items	TMR	Rice Straw
Ingredients, kg		
Rice straw	44.0	
Cassava chip	27.0	
Rice bran	15.5	
Soybean meal	10.0	
Urea	1.5	
Molasses	1.0	
Salt	0.5	
Mineral mixed *	0.25	
Sulfur powder	0.25	
Chemical composition		
Dry matter (DM, %)	66.3	90.1
	% of dry matter	
Organic matter (OM)	88.1	82.3
Crude protein (CP)	10.5	2.5
Ether extract (EE)	3.2	2.3
Neutral detergent fiber (NDF)	37.6	81.7
Acid detergent fiber (ADF)	20.2	52.6

* Contains per kilogram premix: 10,000,000 IU of vitamin A; 70,000 IU of vitamin E; 1,600,000 IU of vitamin D3 (cholecalciferol); 50 g of iron; 40 g of zinc; 40 g of manganese; 0.1 g of cobalt; 10 g of copper; 0.1 g of selenium; 0.5 g of iodine.

**Table 2:** Chemical composition (% of DM) of fermented total mixed ration (FTMR) affected by fermentation time and MMFL supplemented levels.

	Chemical Composition (% of Dry Matter)				
Variable	Organic Matter	Crude Protein	Ether Extract	Neutral Detergent Fiber	Acid Detergent Fiber
Fermentation time (day)					
0	90.90 ^c^	13.76 ^d^	3.28 ^c^	34.25 ^a^	20.20 ^a^
7	91.39 ^b^	14.08 ^c^	3.35 ^bc^	33.85 ^a^	19.05 ^b^
14	92.24 ^a^	14.59 ^b^	3.59 ^ab^	32.55 ^b^	19.00 ^bc^
21	92.37 ^a^	14.95 ^a^	3.70 ^a^	32.07 ^b^	18.06 ^c^
SEM	0.249	0.191	0.178	0.393	0.273
*p*-value (Fermentation time)	0.001	0.002	0.03	0.009	0.002
MMFL levels (% of feed)					
0.0	91.02 ^C^	12.71 ^D^	3.19 ^B^	33.87 ^A^	19.95 ^A^
0.5	91.66 ^B^	14.19 ^C^	3.48 ^A^	33.05 ^B^	19.10 ^B^
1.0	91.91 ^B^	14.90 ^B^	3.58 ^A^	32.85 ^B^	18.95 ^B^
1.5	92.30 ^A^	15.58 ^A^	3.68 ^A^	32.95 ^B^	18.84 ^B^
SEM	0.249	0.191	0.178	0.393	0.273
*p*-value (MMFL levels)	0.004	0.003	0.007	0.008	0.001
Orthogonal polynomial					
Fermentation time					
Linear	0.001	0.004	0.006	0.001	0.001
Quadratic	0.21	0.23	0.98	0.88	0.02
Cubic	0.07	0.34	0.88	0.06	0.04
MMFL levels					
Linear	0.002	0.001	0.003	0.001	0.003
Quadratic	0.98	0.007	0.11	0.03	0.04
Cubic	0.12	0.21	0.55	0.41	0.99

^a,b,c,d^ Value on the same column with different superscripts differ for fermentation time (*p* < 0.05), ^A,B,C,D^ Value on the same column with different superscripts differ for MMFL levels (*p* < 0.05), SEM = Standard error of the mean, MMFL = Mixed microbes fermented liquid. Reported *p*-values represent significance of main effects and the interaction (Fermentation time × MMFL level).

**Table 3:** Effects of mixed microbes fermented liquid (MMFL) ensiled in fermented total mixed ration (FTMR) on gas kinetics, cumulative gas at 72 h after incubation, and in vitro digestibility at 96 h after incubation for different combinations of fermentation time and MMFL levels.

Fermentation Time (Day)	MMFL Levels	Gas Kinetics ^2^				Gas (72 h) mL/0.5 g DM Substrate	In Vitro Degradability ^3^ (%)	
		a (mL)	b (mL)	a (h^−1^)	a + b (mL)		IVDMD	IVOMD
0	0	–0.02 ^d^	40.99 ^d^	0.069 ^b^	40.97 ^d^	40.76 ^d^	57.27 ^d^	88.21 ^e^
	0.5	–1.71 ^cd^	69.49 ^c^	0.083 ^ab^	67.77 ^b^	69.03 ^c^	67.15 ^c^	94.22 ^b^
	1	–0.73 ^cd^	52.47 ^c^	0.073 ^b^	51.73 ^c^	52.46 ^c^	45.76 ^e^	92.43 ^b^
	1.5	–1.61 ^cd^	60.50 ^c^	0.076 ^b^	58.88 ^b^	59.46 ^c^	60.16 ^d^	88.60 ^d^
7	0	–1.76 ^cd^	77.88 ^b^	0.077 ^ab^	76.03 ^ab^	76.47 ^b^	63.24 ^c^	87.43 ^d^
	0.5	–2.26 ^cd^	78.23 ^b^	0.090 ^ab^	75.97 ^ab^	76.07 ^b^	63.06 ^c^	93.04 ^b^
	1	–3.14 ^bc^	83.16 ^a^	0.084 ^ab^	80.02 ^a^	80.25 ^ab^	66.01 ^c^	95.60 ^a^
	1.5	0.04 ^d^	57.17 ^c^	0.076 ^b^	57.21 ^c^	57.90 ^c^	69.25 ^b^	91.48 ^c^
14	0	–4.17 ^b^	75.30 ^b^	0.098 ^a^	71.13 ^ab^	70.66 ^b^	60.62 ^c^	88.94 ^d^
	0.5	–3.77 ^b^	90.53 ^a^	0.100 ^a^	86.75 ^a^	87.40 ^a^	83.34 ^a^	96.45 ^a^
	1	–3.43 ^b^	79.31 ^ab^	0.097 ^a^	75.88 ^ab^	76.03 ^b^	80.11 ^a^	95.67 ^a^
	1.5	–3.42 ^b^	73.83 ^b^	0.092 ^ab^	70.40 ^b^	69.66 ^b^	79.33 ^a^	94.16 ^b^
21	0	–1.89 ^cd^	49.48 ^c^	0.071 ^b^	47.58 ^c^	47.36 ^c^	64.22 ^c^	90.64 ^c^
	0.5	–2.59 ^cd^	70.83 ^b^	0.087 ^ab^	68.24 ^b^	68.64 ^b^	65.50 ^c^	96.40 ^a^
	1	0.07 ^d^	58.08 ^c^	0.091 ^ab^	58.16 ^c^	59.10 ^c^	75.10 ^b^	94.75 ^b^
	1.5	–2.81 ^cd^	62.56 ^c^	0.096 ^ab^	59.75 ^c^	59.75 ^c^	60.35 ^c^	86.50 ^e^
SEM		0.143	0.842	0.0009	0.795	0.785	0.331	0.053
Effects								
Fermentation time		0.002	0.004	0.001	0.003	0.005	0.001	0.003
MMFL levels		0.23	0.004	0.005	0.002	0.007	0.003	0.008
Fermentation time × MMFL		0.008	0.009	0.003	0.004	0.005	0.001	0.002
Orthogonal polynomial								
Fermentation time								
Linear		0.001	0.02	0.009	0.02	0.12	0.01	0.008
Quadratic		0.009	0.004	0.003	0.001	0.009	0.006	0.007
Cubic		0.002	0.23	0.007	0.008	0.001	0.008	0.003
MMFL levels								
Linear		0.12	0.21	0.99	0.13	0.89	0.03	0.001
Quadratic		0.87	0.003	0.004	0.008	0.003	0.008	0.007
Cubic		0.66	0.001	0.05	0.006	0.002	0.04	0.005

^a–e^ Value on the same column with different superscripts differ significantly (*p* < 0.05) among the 16 treatment combinations of fermentation time (0, 7, 14, 21 days) and MMFL levels (0, 0.5, 1.0, 1.5%), SEM = standard error of the mean. Reported *p*-values represent significance of main effects, interaction (Fermentation time × MMFL), and orthogonal polynomial contrasts, ^2^ a = The gas production from the immediately soluble fraction, b = The gas production from the insoluble fraction, c = The gas production rate constant for the insoluble fraction (b), a + b = The gas potential extent of gas production. ^3^ IVDMD = in vitro dry matter degradability, IVOMD = in vitro organic matter degradability.

**Table 4:** Effects of fermentation time and MMFL level ensiled in fermented total mixed ration (FTMR) on volatile fatty acids concentration (VFAs), ammonia nitrogen (NH_3_-N), and methane estimation (CH_4_).

Fermentation Time (Days)	MMFL Levels ^1^	Total VFA (mmol/L)	Acetate (C_2_) (%)	Propionate (C_3_) (%)	Butyrate (C_4_) (%)	C_2_:C_3_ Ratio	NH_3_-N (mg/dL)	CH_4_ Estimation (mmol/L)
0	0.0	47.16 ^d^	71.56 ^a^	15.75 ^c^	12.66 ^a^	4.59 ^a^	18.57 ^ab^	32.92 ^a^
	0.5	51.89 ^cd^	67.30 ^ab^	21.38 ^b^	11.32 ^ab^	3.17 ^b^	18.25 ^bc^	28.93 ^bc^
	1.0	51.24 ^cd^	68.79 ^ab^	18.78 ^b^	12.43 ^a^	3.68 ^ab^	17.73 ^bc^	30.77 ^b^
	1.5	47.94 ^d^	69.37 ^ab^	18.01 ^b^	12.54 ^a^	3.88 ^ab^	17.88 ^bc^	31.32 ^b^
7	0.0	50.12 ^cd^	66.38 ^bc^	21.59 ^b^	12.04 ^ab^	3.08 ^b^	17.54 ^bc^	28.75 ^bc^
	0.5	55.60 ^bc^	63.03 ^c^	26.05 ^a^	10.93 ^b^	2.43 ^c^	19.36 ^ab^	27.53 ^c^
	1.0	55.05 ^bc^	65.90 ^bc^	23.36 ^ab^	10.75 ^b^	2.82 ^c^	19.27 ^ab^	27.37 ^c^
	1.5	53.55 ^bc^	65.79 ^bc^	23.10 ^ab^	11.12 ^b^	2.85 ^c^	18.64 ^bc^	27.70 ^c^
14	0.0	57.72 ^bc^	58.20 ^d^	30.19 ^a^	11.62 ^b^	2.00 ^c^	18.60 ^bc^	22.53 ^d^
	0.5	59.36 ^ab^	56.00 ^d^	33.41 ^a^	10.59 ^b^	1.68 ^c^	20.14 ^ab^	20.26 ^d^
	1.0	58.98 ^ab^	58.85 ^cd^	31.17 ^a^	9.99 ^b^	1.89 ^c^	19.73 ^ab^	21.91 ^d^
	1.5	58.99 ^ab^	56.81 ^d^	31.79 ^a^	11.41 ^b^	1.79 ^c^	19.38 ^ab^	21.39 ^d^
21	0.0	51.11 ^cd^	62.30 ^c^	25.97 ^ab^	11.74 ^b^	2.40 ^c^	18.18 ^bc^	25.59 ^c^
	0.5	56.67 ^bc^	62.75 ^c^	27.61 ^ab^	9.65 ^b^	2.27 ^c^	18.70 ^bc^	24.50 ^c^
	1.0	56.44 ^bc^	61.47 ^c^	27.53 ^ab^	11.01 ^b^	2.23 ^c^	18.55 ^bc^	24.49 ^c^
	1.5	56.44 ^bc^	61.47 ^c^	27.53 ^ab^	11.01 ^b^	2.23 ^c^	18.55 ^bc^	24.49 ^c^
SEM		2.066	1.635	1.621	1.307	0.263	2.066	1.150
Effects								
Fermentation time		0.001	0.009	0.008	0.23	0.001	0.09	0.001
MMFL levels		0.02	0.99	0.04	0.99	0.01	0.11	0.01
Fermentation time × MMFL		0.34	0.53	0.12	0.99	0.78	0.81	0.67
Orthogonal polynomial								
Fermentation time								
Linear		0.001	0.009	0.008	0.22	0.007	0.89	0.003
Quadratic		0.001	0.004	0.008	0.66	0.006	0.04	0.002
Cubic		0.04	0.005	0.007	0.56	0.02	0.91	0.003
MMFL levels								
Linear		0.04	0.90	0.78	0.77	0.67	0.97	0.55
Quadratic		0.02	0.88	0.04	0.88	0.02	0.03	0.03
Cubic		0.99	0.96	0.057	0.55	0.089	0.56	0.056

^a–d^ Value on the same column with different superscripts differ significantly (*p* < 0.05) among the 16 treatment combinations of fermentation time (0, 7, 14, 21 days) and MMFL levels (0, 0.5, 1.0, 1.5%), SEM = standard error of the mean. Reported *p*-values represent significance of main effects, interaction (Fermentation time × MMFL), and orthogonal polynomial contrasts, ^1^ Mixed microbes fermented liquid (MMFL). Methane was estimated using the stoichiometric equation of Moss et al. based on individual VFA concentrations (mmol/L), calculated from total VFA concentration and molar proportions of acetate, propionate, and butyrate.

**Table 5:** Effects of fermentation time and MMFL levels on ruminal microorganism population.

		Total Direct Count (Cell/mL)		
Fermentation Time (Days)	MMFL Levels ^1^	Bacteria (×10^7^)	Protozoa (×10^5^)	Fungal Zoospores (×10^5^)
0	0.0	2.95 ^d^	6.77 ^a^	2.75 ^d^
	0.5	4.28 ^c^	5.71 ^b^	4.15 ^b^
	1.0	3.93 ^c^	5.96 ^b^	3.29 ^c^
	1.5	3.63 ^c^	6.06 ^b^	3.35 ^c^
7	0.0	3.43 ^c^	6.04 ^b^	3.48 ^c^
	0.5	4.35 ^c^	4.56 ^c^	5.30 ^a^
	1.0	4.10 ^c^	5.23 ^b^	4.00 ^b^
	1.5	3.97 ^c^	5.40 ^b^	3.90 ^b^
14	0.0	4.85 ^b^	5.00 ^b^	4.25 ^b^
	0.5	5.85 ^a^	3.85 ^c^	5.70 ^a^
	1.0	5.15 ^ab^	4.10 ^c^	5.20 ^a^
	1.5	5.20 ^ab^	4.10 ^c^	4.85 ^ab^
21	0.0	3.85 ^c^	6.25 ^b^	3.75 ^b^
	0.5	5.50 ^ab^	4.45 ^c^	5.05 ^a^
	1.0	4.95 ^b^	5.45 ^b^	4.20 ^b^
	1.5	5.20 ^ab^	5.44 ^b^	4.00 ^b^
SEM		0.352	0.453	0.435
Effects				
Fermentation time		0.001	0.008	0.009
MMFL levels		0.004	0.008	0.002
Fermentation time × MMFL		0.89	0.67	0.44
Orthogonal polynomial				
Fermentation time				
Linear		0.001	0.007	0.006
Quadratic		0.08	0.002	0.007
Cubic		0.002	0.02	0.23
MMFL Levels				
Linear		0.04	0.88	0.89
Quadratic		0.003	0.009	0.007
Cubic		0.03	0.04	0.004

^a–d^ Value on the same column with different superscripts differ significantly (*p* < 0.05) among the 16 treatment combinations of fermentation time (0, 7, 14, 21 days) and MMFL levels (0, 0.5, 1.0, 1.5%), SEM = standard error of the mean. Reported *p*-values represent significance of main effects, interaction (Fermentation time × MMFL), and orthogonal polynomial contrasts, ^1^ Mixed microbes fermented liquid (MMFL).

## Data Availability

The original data presented in this study are included in the article. Further inquiries can be directed to the corresponding author(s).
